# Relationship between Cardiopulmonary, Mitochondrial and Autonomic Nervous System Function Improvement after an Individualised Activity Programme upon Chronic Fatigue Syndrome Patients

**DOI:** 10.3390/jcm10071542

**Published:** 2021-04-06

**Authors:** Sławomir Kujawski, Jo Cossington, Joanna Słomko, Monika Zawadka-Kunikowska, Małgorzata Tafil-Klawe, Jacek J. Klawe, Katarzyna Buszko, Djordje G. Jakovljevic, Mariusz Kozakiewicz, Karl J. Morten, Helen Dawes, James W. L. Strong, Modra Murovska, Jessica Van Oosterwijck, Fernando Estevez-Lopez, Julia L. Newton, Lynette Hodges, Paweł Zalewski, on behalf of the European Network on ME/CFS (EUROMENE)

**Affiliations:** 1 Department of Hygiene, Epidemiology, Ergonomy and Postgraduate Education, Ludwik Rydygier Collegium Medicum in Bydgoszcz, Nicolaus Copernicus University in Torun, M. Sklodowskiej-Curie 9, 85-094 Bydgoszcz, Poland; jslomko@cm.umk.pl (J.S.); m.zkunikowska@cm.umk.pl (M.Z.-K.); jklawe@cm.umk.pl (J.J.K.); p.zalewski@cm.umk.pl (P.Z.); 2 Centre for Movement Occupational and Rehabilitation Sciences, Department of Sport, Health Sciences and Social Work, Oxford Brookes University, Headington Rd, Headington, Oxford OX3 0BP, UK; jcossington@brookes.ac.uk (J.C.); hdawes@brookes.ac.uk (H.D.); 3 Department of Human Physiology, Ludwik Rydygier Collegium Medicum in Bydgoszcz, Nicolaus Copernicus University in Torun, Karłowicza 24, 85-092 Bydgoszcz, Poland; malg@cm.umk.pl; 4 Department of Biostatistics and Biomedical Systems Theory, Collegium Medicum, Nicolaus Copernicus University, Jagiellonska Street, 85–067 Bydgoszcz, Poland; buszko@cm.umk.pl; 5 Institute of Health and Wellbeing, Faculty of Health and Life Sciences, Priory St, Coventry CV1 5FB, UK; djordje.jakovljevic@coventry.ac.uk; 6 Department of Geriatrics, Ludwik Rydygier Collegium Medicum in Bydgoszcz, Nicolaus Copernicus University in Torun, M. Sklodowskiej-Curie 9, 85-094 Bydgoszcz, Poland; markoz@cm.umk.pl; 7 Nuffield Department of Women’s & Reproductive Health, The Women Centre, University of Oxford, Oxford OX3 9DU, UK; karl.morten@wrh.ox.ac.uk (K.J.M.); jamie.strong@kellogg.ox.ac.uk (J.W.L.S.); 8 NIHR Oxford Health Biomedical Research Centre, Oxford OX3 7JX, UK; 9 Institute of Microbiology and Virology, Riga Stradiņš University, LV-1067 Riga, Latvia; Modra.Murovska@rsu.lv; 10 Department of Rehabilitation Sciences, Ghent University, 9000 Ghent, Belgium; Jessica.VanOosterwijck@UGent.be; 11 Research Foundation—Flanders (FWO), 1000 Brussels, Belgium; 12 Department of Child and Adolescent Psychiatry/Psychology, Erasmus MC University Medical Center, Postbus 2060, 3000 CB Rotterdam, The Netherlands; fer@estevez-lopez.com; 13 Population Health Sciences Institute, The Medical School, Newcastle University, Newcastle-upon-Tyne NE2 4AX, UK; julia.newton@ncl.ac.uk; 14 School of Sport, Exercise and Nutrition, Massey University, Palmerston North 4442, New Zealand; L.D.Hodges@massey.ac.nz

**Keywords:** myalgic encephalomyelitis, chronic fatigue syndrome, autonomic nervous system, exercise, mitofusin, oxygen consumption

## Abstract

Background: The therapeutic effects of exercise from structured activity programmes have recently been questioned; as a result, this study examines the impact of an Individualised Activity Program (IAP) on the relationship with cardiovascular, mitochondrial and fatigue parameters. Methods: Chronic fatigue syndrome (CFS) patients were assessed using Chalder Fatigue Questionnaire (CFQ), Fatigue Severity Score (FSS) and the Fatigue Impact Scale (FIS). VO_2_peak, VO_2_submax and heart rate (HR) were assessed using cardiopulmonary exercise testing. Mfn1 and Mfn2 levels in plasma were assessed. A Task Force Monitor was used to assess ANS functioning in supine rest and in response to the Head-Up Tilt Test (HUTT). Results: Thirty-four patients completed 16 weeks of the IAP. The CFQ, FSS and FIS scores decreased significantly along with a significant increase in Mfn1 and Mfn2 levels (*p* = 0.002 and *p* = 0.00005, respectively). The relationships between VO_2_ peak and Mfn1 increase in response to IAP (*p* = 0.03) and between VO_2_ at anaerobic threshold and ANS response to the HUTT (*p* = 0.03) were noted. Conclusions: It is concluded that IAP reduces fatigue and improves functional performance along with changes in autonomic and mitochondrial function. However, caution must be applied as exercise was not well tolerated by 51% of patients.

## 1. Introduction

Chronic Fatigue Syndrome (CFS) is a complex condition characterised by symptoms including chronic fatigue, disturbance in cognitive functions, autonomic dysfunction, pain, ineffective sleep and exercise intolerance [1,2]. Physical or mental exertion might lead to intense debilitating fatigue, musculoskeletal pain, sleep disturbance, headaches, impairments in concentration and short-term memory [3]. Accumulating evidence suggests that the cardiovascular system may be compromised in individuals suffering from CFS, along with reports of autonomic dysfunction [3], impaired heart rate (HR), blood pressure regulation and impaired heart conduction [4].

Autonomic nervous system (ANS) dysfunction is one of the widely described parts of CFS pathomechanism [4]. ANS function can be measured non-invasively using heart rate variability (HRV) [5,6], which differentiates between healthy and diseased states, and is associated with mortality [7]. HRV seems to be a useful biomarker of mental health, stress response and adaptation [8].

Based on HRV, it is proposed that low frequency (LF) variability is an indicator of sympathetic nervous system activity, while high frequency (HF) is an indicator of vagal activity [5,6]. A recent meta-analysis suggests that resting sympathetic hyperactivity, indicated by changes in HRV and blood pressure variability (BPV) might be related to a lower HRmax in CFS patients compared to healthy controls [4]. It is anticipated that chronic sympathetic overactivity might lead to the downregulation of ANS receptors and therefore may suppress HRmax. Moreover, the HR response to a head-up tilt testing (HUTT) was higher in CFS patients compared to healthy controls [4]. The HUTT might serve as a tool in the diagnosis of ANS dysfunction [9,10]. Whilst physical activity programmes might lead to improvement in the ANS in athletes [11], low-volume high-intensity training also improves HRV in sedentary adult men [12]. In addition, the relationship between increased VO2max and increased HRV has also been observed in response to a physical activity program [13].

Although fatigue is multi-dimensional in nature it was recently demonstrated that VO_2_peak could be an independent predictor of fatigue [14] thus, suggesting the importance of measuring this as a component of health-related fatigue. A recent meta-analysis [15] compared the data of healthy controls and those with CFS from a single exercise test. Franklin et al. [15] demonstrated a pooled mean VO_2_peak that was 5.2 mL kg^−1^ min^−1^ lower in CFS compared to healthy controls [15]. However, between subject variability was 3.5 (1.5–4.5 mL kg^−1^ min^−1^) indicating substantial heterogeneity.

In addition to ANS disturbance, mitochondrial dysfunction may also be present in individuals with CFS [16]. The examination of biopsy of muscle tissue using electron microscopy has shown degeneration of mitochondria within this population [17,18,19]. Healthy mitochondria undergo continual fusion that requires GTPase transmembrane proteins mitofusin 1 (Mfn1) and mitofusin 2 (Mfn2). Deletion of Mfn1 and Mfn2 can lead to a decrease in exercise capacity, which is brought about and worsened by dysfunction in Complex I and IV [20]. Both Mfn1 and 2 are upregulated after physical exercise training in healthy individuals [21]. During starvation or stress, mitochondria form a network structure with mitochondria in a fused state [22]. In this state it is proposed that mitochondria are more efficient at making ATP when substrates are limiting sharing respiratory chain complexes and making the best use of substrates available [23,24,25]. However, more recently mitochondrial hyperfusion has been linked to various diseases with a negative effect on cell function (reviewed in Rajdeep [26]). Endurance training has the potential to increase mitochondrial functioning, improving biogenesis, mitophagy, and efficiency altering fusion and fission [27]. As already has been mentioned, physical activity programmes might lead to improvement of ANS in healthy participants [12,27]. Therefore, as disturbance in bioenergetics and ANS might be a presumably important parts of CFS pathogenesis, physical exercise program could be applied in patients to improve function of those systems. 

Previous studies have documented that a structured activity programme for CFS could be beneficial in some patients in terms of fatigue and disabilities [28,29,30]. Nevertheless, the therapeutic effectiveness of aerobic physical exercise programmes on CFS seems to be unclear and controversial [31,32]. What seems important is that long-term efficacy of physical exercise programs in CFS patients has been disputed with lack of significant improvement in fatigue and disability compared to patients allocated to receiving standard medical care (SMC) for at least 2 years follow-up [31,32]. Importantly, only four percent of patients from GET group could be considered to be “recovered” when an intention-to-treat approach and protocol-specified definition of recovery is applied [31]. Moreover, long term changes in the examined groups were not statistically significant [31].

This study examines the effects of an Individualised Activity Program (IAP) on self-reported fatigue, respiratory (VO_2_sumbax and VO_2_peak), ANS (low frequency to high frequency ratio of R-R interval (LF/HF-RRI) at rest and during HUTT) and mitochondrial (Mfn1 and Mfn2 levels) functioning in CFS patients and the interaction of these outcomes to provide more insight into the disturbance in underlying mechanisms of the exercise effects.

## 2. Materials and Methods

An activity-based study was performed, which included a homebased exercise intervention and two testing visits, one at baseline, and one post exercise intervention. The patients’ progress was supervised during telephone calls which took place every week. The study was approved by the Ethics Committee, Ludwik Rydygier Memorial Collegium Medicum in Bydgoszcz, Nicolaus Copernicus University, Torun, Poland (KB 332/2013, date of approval: 25 June 2013) and written informed consent was obtained from all participants.

### 2.1. Recruitment and Eligibility

**Figure 1:**
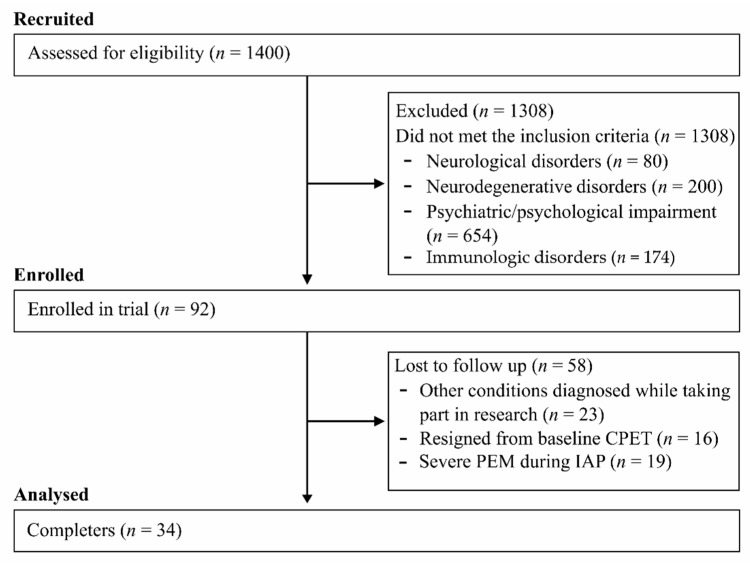
CONSORT-type flow diagram.

CFS patients were included if they met the diagnostic criteria of the Fukuda case definition for CFS [33]. The patients were recruited based on advertisements in both local and national TV and newspapers. Initially, 1400 volunteers were assessed for eligibility onto the trial with 1308 being excluded. Neurological (myasthenia gravis, traumatic brain injury, stroke, etc.), neurodegenerative (Parkinson’s disease, multiple sclerosis, amyotrophic lateral sclerosis, etc.), psychiatric/psychological impairment (atypical depression, generalized anxiety disorder, etc.) and immunologic disorders (systemic lupus erythematosus, type 1 diabetes, celiac disease, rheumatoid arthritis, etc.) which were excluding factors comprised those of which mechanisms might presumably explain primary symptoms of CFS (reasons for exclusion depicted in Figure 1). This left 69 individuals who met the trial inclusion criteria. However, only 53 patients were willing to partake and follow the IAP protocol. Sixteen CFS patients chose not to undertake baseline cardiopulmonary exercise test (CPET). Nineteen patients dropped out due to reported severe post-exertional malaise (PEM) reaction to the IAP [34]. The recruitment and participant flow through the study is shown in Figure 1. A control group was not recruited in the above study. 

#### Anxiety and Depression

A Hospital Anxiety and Depression Scale (HADS) [35] was performed to assess anxiety (HADS_A) and depression (HADS_D) symptoms intensity. Beck Depression Inventory (BDI-II) was used to examine depression symptoms intensity [36]. Both scales were used only at the baseline to exclude patients with depression.

### 2.2. Outcome Measures

#### 2.2.1. Body Composition Analysis

To measure body composition changes a multi frequency bioelectrical impedance analyser (Tanita MC-180MA Body Composition Analyzer, Tanita UK Ltd., Manchester, UK) was applied. All subjects were attributed a ‘normal’ proprietary algorithm for the impedance measurement. Before measurement, the soles of the feet and the inner part of the hand were cleaned with a sterile dressing to remove any lipid layer. Subjects stood with the ball and heel of each foot in contact with the electrodes on the floor scale. After recording weight in kilograms, the subjects grasped the hand grips with electrode and held them down by their sides with arms extended and away from the body to continue body composition analysis based on bioelectrical impedance signal. Weight kilograms and height in centimetres were measured, and body mass index (BMI) was calculated as well as percent of fat mass and free-fat mass (FFM) in kilograms.

#### 2.2.2. Fatigue

The Chalder Fatigue Questionnaire (CFQ) [37], Fatigue Severity Score (FSS) [38] and the Fatigue Impact Scale (FIS) [39] were administered to provide a comprehensive assessment of fatigue severity. The CFQ assessed physical and psychological fatigue, FSS assessed fatigue in the past week and the FIS assessed cognitive, physical and psychosocial fatigue. Higher scores indicate higher severity in all domains. All questionnaires were administered at baseline and post intervention. 

#### 2.2.3. Autonomic Nervous System (ANS) Functioning

ANS functioning was measured with a Task Force Monitor—TFM (CNS Systems, Gratz, Austria). Signals from three-channel ECG were analysed using the adaptive autoregressive model [40]. Low frequency (LFnu-RRI) (0.04–0.15 Hz) and high frequency (HFnu-RRI) (0.15–0.4 Hz) components of R to R intervals in normalized units as well as its ratio (LF/HR-RRI) were recorded and analysed in rest and in response to HUTT. Assessments were performed after 5 min waiting period in supine position which allowed for signals to stabilize. Then, an assessment at rest was performed in supine position which lasted for another 5 min. Heart rate (HR), systolic blood pressure (sBP), diastolic blood pressure (dBP) were measured during rest. Moreover, cardiac index (CI) which is a cardiac output from left ventricle in one minute in relation to body surface area (BSA) was assessed based on cardioimpendance signal. Afterwards, the assessment was performed during a passive HUTT at 70° angle of inclination following the Newcastle protocol [41]. The duration of the HUTT was six minutes which is in line with previous reports [42]. A tilt table with foot support and fastening straps at the knee, hip and chest levels was used to passively change the body position. Differences between mean values of parameters during the third to fourth minute of HUTT along with mean values from the supine position were analysed. 

#### 2.2.4. Mitochondrial Function

Blood samples were taken before (baseline) and after IAP (at 16 weeks) to perform biochemical analysis. For plasma, whole blood was collected into commercially available (Vacutainer) anticoagulant EDTA-treated (lavender tops). The cells were removed from plasma by centrifugation for 15 min at 2500× *g* using a refrigerated (+4 °C) centrifuge. The resulting supernatant was designated as plasma. After centrifugation plasma was immediately transferred into a clean sterilized polypropylene tube. The samples were stored at −80 °C until the analysis. Patients started IAP in a sequential manner, therefore some samples of initially recruited patients were frozen longer than the other samples. All samples were defrosted and analysed together. Mfn1and Mfn2 levels were examined using enzyme-linked immunosorbent assay (ELISA) tests (Cloud-Clone, Katy, TX, USA). 

#### 2.2.5. Cardiorespiratory Function

In the presence of a physician, the patients undertook a cardiopulmonary exercise test (CPET) on a treadmill using the Bruce protocol at baseline and at post intervention [43]. A trained technician provided brief instructions and advised the test would end at the moment of full exertion, on the command of the physician, or at any other time point, as stated by the guidelines for safe exercise testing by the American College of Sports Medicine [44]. During exercise there was continuous cardiorespiratory monitoring (Cardiovit CS-200 Ergo-Spiro, Schiller AG, Baar, Switzerland). Heart rate (HR, VO_2_, load (watt) and respiratory exchange ratio (RER (VCO_2_:VO_2_)) were measured to assess cardiopulmonary fitness at baseline and after the intervention. The anaerobic threshold (AT) was determined using the V-slope method [45].

### 2.3. Intervention

#### Individualised Activity Programme (IAP)

The IAP has been previously reported [34] and consisted of a prescribed 16-week multimodal home activity programme. The activities were performed 5 days a week, with time (10–40 min) and intensity (30–80% HRpek) increasing gradually across the time period. The HR intensity during activity was individually prescribed based on the actual HRpeak achieved during the CPET. Patients were equipped with HR monitors (Beurer PM 25) to help them in sustaining the recommended HR. Every week, telephone calls were made to resolve potential problems with compliance and to ensure patients were satisfied with the protocol. Patients underwent a minimum of 80 activity sessions, which was the total number of sessions in 16 weeks.

### 2.4. Statistical Methods

Mitofusins level were not assessed in one patient due to technical difficulties during blood drawing (both before and after physical exercise program) and therefore data on mitofusins level from this patient was not included into analysis. Descriptive statistics include the calculation of means and standard deviations. The Shapiro–Wilk test was used to test the assumption of normality. Variables where values did not meet the normality of distribution assumption, were analysed using Wilcoxon signed-rank test, which was used to compare pre vs. post intervention outcomes. In all other cases t-tests of dependent samples were used. R denotes effect size for Wilcoxon signed-rank test and student t-test provided for statistically significant results [46]. The above tests were performed using statistical package STATISTICA 13.1 (StatSoft, Inc., Tulsa, OK, USA).

Mixed models with random effects were applied to analyse the dependence of CFS, FSS and FIS scales on the CPET and ANS indicators measured at rest and in response to the HUTT. In order to assess the dependence of cardiopulmonary functioning on biochemical parameters and ANS indicators measured at rest and changes in response to tilt mixed models with random effects was performed. In each model, the patients’ effects were fitted as random. In the models the maximum likelihood method was applied for estimating variance parameters. Analyses were performed with R version 3.6.2 (R: library lme) [47]. Spearman’s correlation was used to analyse relationship between outcomes of the study.

Graphs were created using an R environment [47] with a ggpubr package based on ggplot2 [48]. Benjamini-Hochberg Adjusted P value was chosen to control for False Discovery Rate (FDR). An online calculator for FDR corrections was used (https://www.sdmproject.com/utilities/?show=FDR, accessed on 17 August 2020). *p*-values prior and following FDR correction are reported.

## 3. Results

Thirty-four CFS patients (20 females, 14 males) completed IAP. Unfortunately, it was not possible for all patients to reach 80% of their HRpeak, and only 1 patient reached this during the last training session. However, 32 patients were able to reach 70% HRmax, and 1 patient achieved 60%HRpeak during the last training sessions. Although patients were encouraged to undertake walking, participants also carried out additional activities including cycling or swimming. All patients chose to perform walking exercises. The mean compliance rate was 80%. Compliance rates for the structured exercise programme were above 60%, which was set as the threshold value. A further examination of the characteristics of IAP completers can be found in Table 1 and Table S1.

### 3.1. Influence of IAP on Fatigue

**Figure 2:**
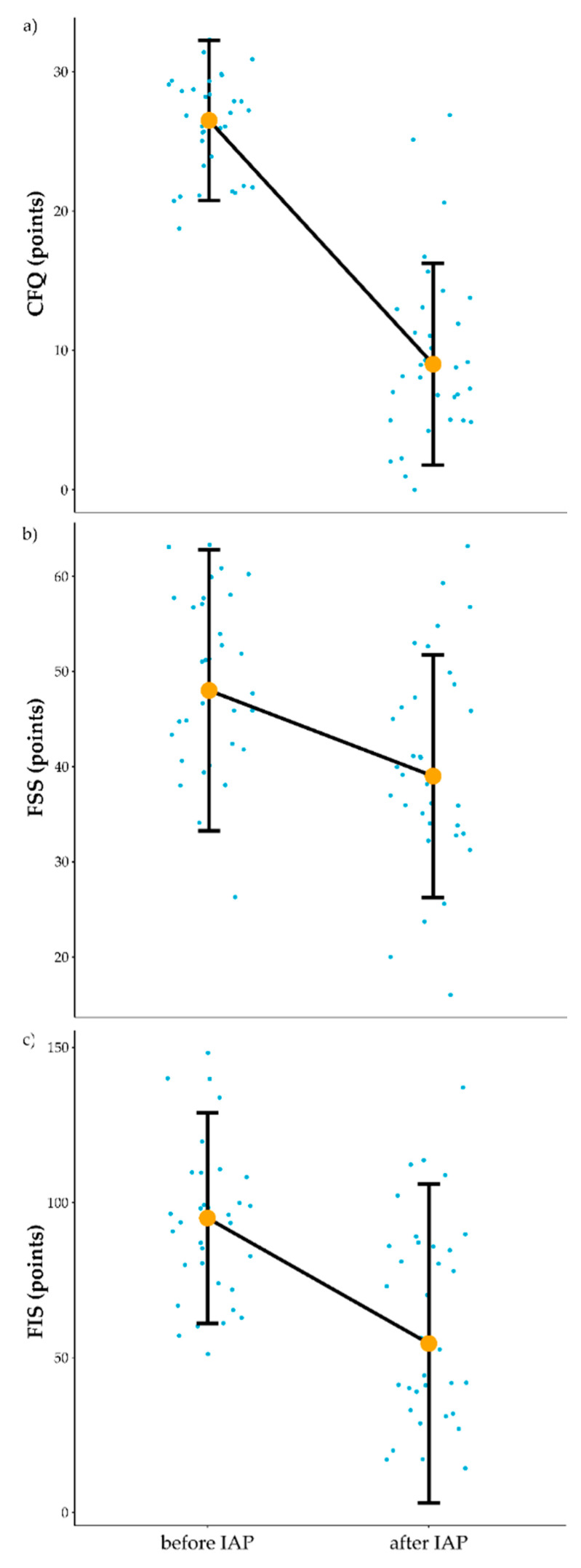
Influence of IAP on fatigue scales. (**a**) influence of IAP on CFS—Chronic Fatigue Scale. (**b**) influence of IAP on FSS—Fatigue Severity Scale. (**c**) influence of IAP on FIS—Fatigue Impact Scale. IAP—Individualised Activity Program, Orange dots connected by black line indicate median value, vertical black lines denote interquartile range. Blue dots before and after denote results of individual patients.

The influence of the intervention on fatigue was the main area of interest in this study. The structured IAP reduced fatigue levels of the patients in a statistically significant manner on all three scales. The mean scores on the CFQ decreased from 26.12 at baseline to 9.68 post intervention (Z = 5.09, *p* < 0.001, r = 0.62) (Figure 2a). The mean scores on the FSS decreased from 48.91 at baseline to 40.15 post intervention (t = 4.66, *p* < 0.0001, r = 0.63) (Figure 2b). Mean scores on the FIS decreased from 93.59 at baseline to 61.68 post intervention (t = 6.75, *p* < 0.0001, r = 0.76) (Figure 2c) (Table S2).

### 3.2. Influence of IAP on Cardiorespiratory Function

**Figure 3:**
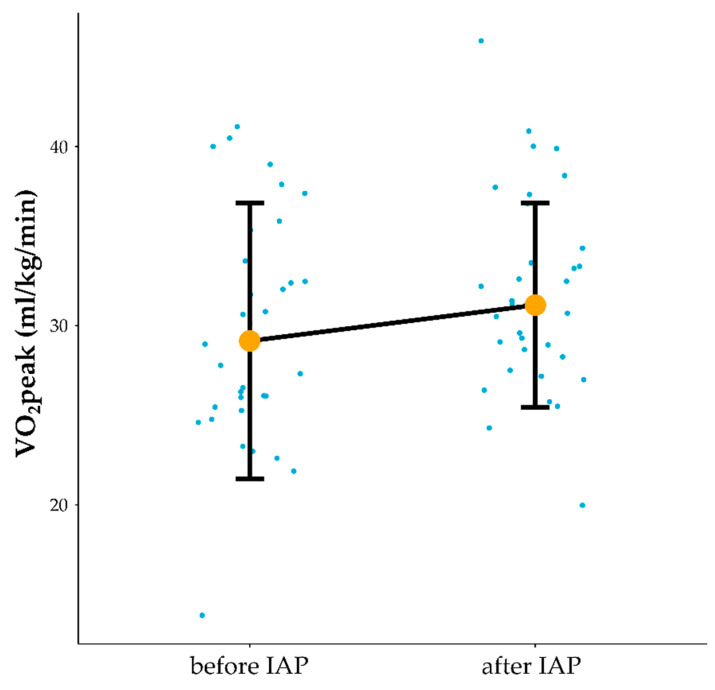
Influence of IAP on VO_2_peak. VO_2_peak mL/kg/min—maximal oxygen uptake during physical exercise measured in ml/kg/min, IAP—Individualised Activity Program, Orange dots connected by black line indicate median value, vertical black lines denote interquartile range. Blue dots before and after denote the results of individual patients.

The impact of the intervention on cardiorespiratory function was important in terms of both the occurrence of positive adaptation to the program and indirect evidence of programme compliance. After IAP treadmill workload normalised to body weight at AT was significantly increased (1.31 W/kg before vs. 1.61 W/kg after IAP), t =−4.53, *p* = 0.00007, r = 0.62 and load/body mass at maximal intensity of physical exercise significantly increased (1.85 W/kg before vs. 2.09 W/kg after), Z = 2.83, *p* = 0.005, r = 0.34. VT at AT significantly increased (1.66 L vs. 1.81 after), Z = 2.74, *p* = 0.01, r = 0.33. VO_2_peak increased significantly (30.3 mL/kg/min before vs. 31.79 after), Z = 1.98, *p* = 0.047, r = 0.24 (Figure 3) (Table S3). Regarding individual patients, clinically significant improvement defined as improvement of >1.1 mL/kg/min in VO_2_peak was noted in 19 patients (7 out of 14 males and 12 out of 20 females). The VO_2_peak improved with 1.66 mL/kg/min when the whole group was considered, and both in males and females. A patient who was able to reach 80% HRmax during the last training session and 18 patients who reached 70% HRmax noted VO_2_peak clinically significant improvement. A patient who reached 60% and 14 patients who reached 70% HRmax during the last training sessions did not gained clinically significant improvement in VO_2_peak. 

### 3.3. Influence of IAP on Mitochondrial Function

**Figure 4:**
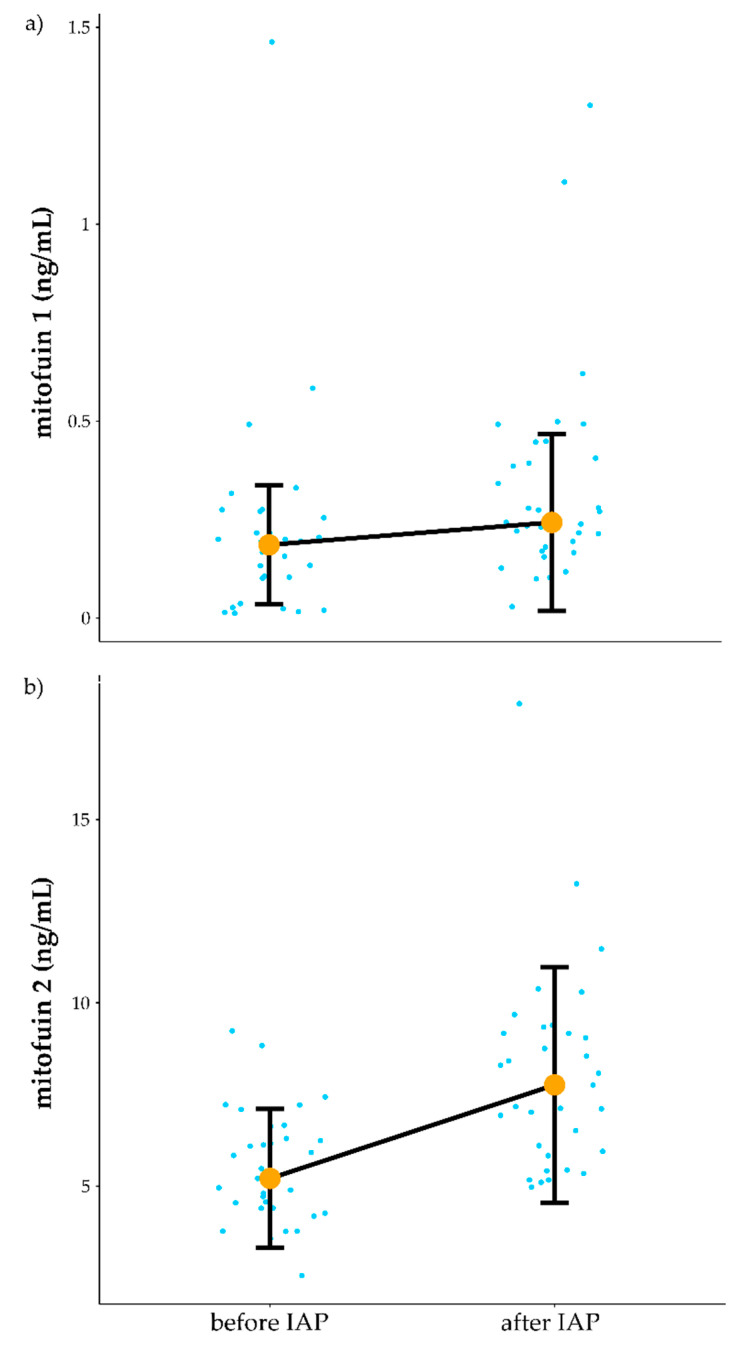
Influence of IAP on Mitofusins. (**a**) influence of IAP on miofusin1 level. (**b**) influence of IAP on mitofusin2 level. IAP—Individualised Activity Program, Orange dots connected by black line indicate median value, vertical black lines denote interquartile range. Blue dots before and after denote the results of individual patients.

Exploring the effects of IAP on mitochondrial function was one aim of this study. Biochemical analyses showed an increase in both plasma Mfn1 and Mfn2 in response to IAP. Mean value of Mfn1 (increased from 0.22 ng/mL before to 0.33 ng/mL after IAP (Z = 3.07, *p* = 0.002, r = 0.38)) (Figure 4a). Moreover, Mfn2 mean value (increased from 5.51 ng/mL before vs. to 8.05 ng/mL following the IAP (Z = 4.06, *p* = 0.00005, r = 0.5) (Figure 4b) (Table S4).

### 3.4. Interaction between VO_2_peak Improvement, Mitochondrial Plasma Markers and ANS Changes

**Figure 5:**
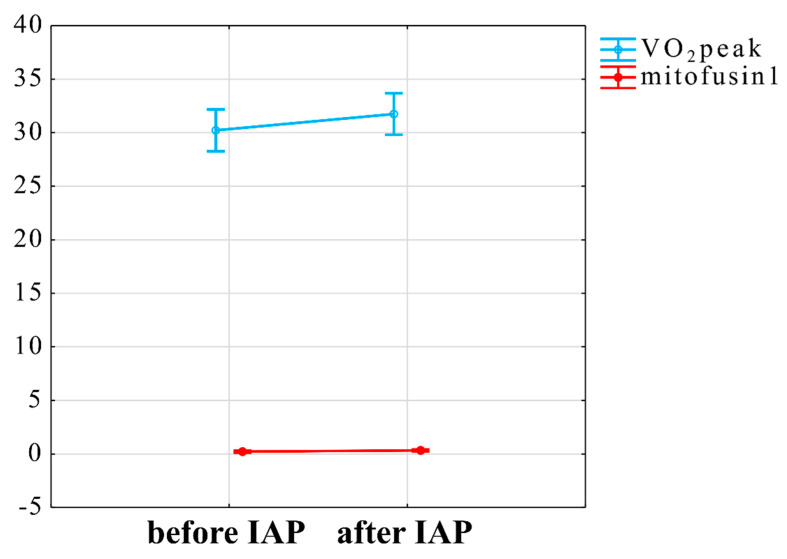
Interaction between influence of IAP on VO_2_peak and Mfn1. VO_2_peak—maximal oxygen consumption obtained during physical exercise, mitofusin1—level of mitofusin1, IAP—Individualised Activity Program.

We explored the role of mitochondria and ANS functions underlying the adaptation to IAP by assessing the indicators of mitochondrial function: MFn1 and MFn2 levels in plasma. The mixed linear model for interaction of VO_2_peak and Mfn1 was statistically significant (t = 2.5, *p* = 0.02) (Figure 5). 

**Figure 6:**
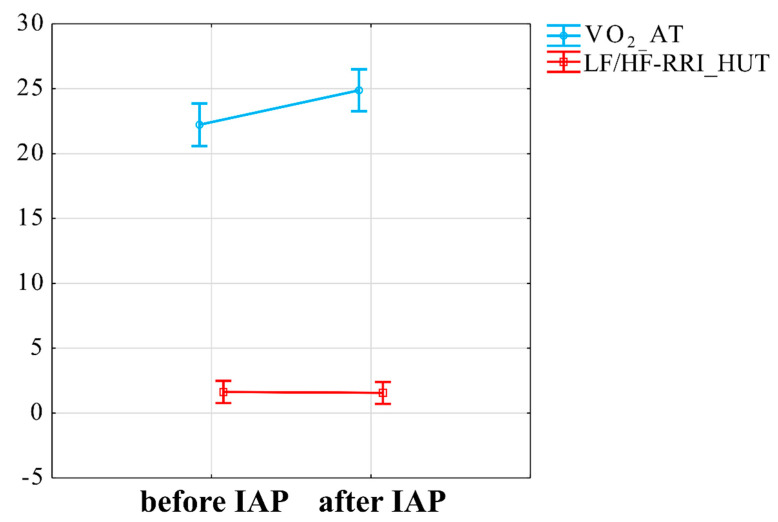
Interaction between influence of IAP on VO_2_subpeak and sympathovagal balance in response to head-up tilt test. VO_2_AT—maximal oxygen consumption during anaerobic threshold, LF/HF-RRI_HUTT—low frequency diastolic blood pressure to high frequency R-R interval during head-up tilt test, IAP—Individualised Activity Program.

Moreover, the interaction between VO_2_ at AT (V-slope method) and LF/HF-RRI change in response to HUTT was also significant (t = −2.05, *p* = 0.048) (Figure 6). Other examined interactions between VO_2_peak, VO_2_subpeak, fatigue scale scores and autonomic outcomes or mitofusins were not significant.

Influence of IAP on autonomic nervous system function indicators was not significant (Table S5). Heatmap of Spearman’s correlation between outcomes of the study is presented on Figure S1.

## 4. Discussion

This study has noted a statistically significant increase in peak VO_2_, alterations in biological factors associated with mitochondria and fatigue in CFS patients who underwent an individualised home-based activity programme. To our knowledge, this is the first study that has associated an increase in maximal aerobic capacity to increased plasma Mfn1 levels. In CFS patients an increase in submax VO_2_ related to a decrease in the ratio of sympathetic to parasympathetic activity during HUTT. In a previous study, we noted that CFS patients who completed the IAP showed improved visual attention both in terms of reaction time and correctness of responses and processing speed of simple visual stimuli [34]. It is important to note that there was a significant drop out rate of 51% with IAP [49]. The more sympathetic drive contributes to the control of blood vessels, the longer the reaction time with simple visual stimuli and the lower the HRmax during physical exercise, the chance of a CFS patient completing IAP was reduced [49]. Overall, it could be concluded that an aerobic activity programme characterised by a high frequency of weekly training sessions (5 times/week) and incremental progression of exercise intensity is not well tolerated by a significant number of CFS patients (51% of the patients in the current study). On the other hand, those able to complete the programme noted a reduction in fatigue and improvement in functional performance at the cognitive and cardiovascular level, the latter change being related to changes in autonomic and mitochondrial markers in plasma. It is therefore clear that careful identification of those most likely to benefit and able to participate is needed. Therefore, it would be advisable that any studies seeking to further ascertain the effects of activity programmes in ME/CFS optimally comprise an appropriate control arm, such as a sham intervention or lower intensity exercise regimen with equal assignment of patients classified as more likely to be able to tolerate an aerobic programme comprising high-frequency training sessions. Moreover, benefits and potential harms of training programmes with lower frequency than 5 sessions per week should be examined in further studies.

### 4.1. Influence of IAP on Self-Reported Fatigue and Peak Oxygen Uptake

The mean CFQ score at baseline was 26.12 in the current study and comparable to 28.2 points scored by the subgroup who received GET in the PACE trial [50]. A 5.4 point decrease in CFQ was previously observed following a 12 week GET trial [51]. The authors of a graded exercise therapy guided self-help trial (GETSET) noted a mean baseline CFQ score of 26.3 for the GET group. After 12-weeks of GET the mean score reduced to 19.1 points, this with a combined effect size of 0.53 [52]. In the current study, the effect size for CFQ scale improvement was 0.62, however, it should be noted that this scale is burdened with many methodological issues which might limit drawing conclusions, especially in longitudinal studies. These issues include problems in the interpretation of the questions when examining the same participants more than once [53]. On the other hand, in our study significant decrease in fatigue were also noted on two other self-reported fatigue measures. The FSS seems to be characterized by high sensitivity and specificity in classification of CFS patients vs. healthy controls [54]. Significant improvement measured by FIS scale was also noted. Hence, these other scales reinforce the observation made using the CFQ assessment. Our study is the first intervention based aerobic activity program in CFS patients that has used three questionnaires to assess the effects on fatigue. 

Using CPET testing peak oxygen uptake levels improved by 1.66 mL/kg/min in IAP completers in the whole group regardless of gender. To be considered clinically important in CFS patients peak oxygen uptake needs to increase by 1.1 mL/kg/min [15], therefore, in this study both male and female completers noted improvements higher than the difference between CFS and healthy controls in peak oxygen uptake. Authors of the PACE trial reported improvement induced by GET in aerobic capacity which was evaluated during a 6-min walk test from 312 m to 379 m at 52-week follow-up. In comparison to the PACE trial, we used CPET, which is the gold standard method to assess physical capacity [51]. In addition, the GETSET study also lacked objective assessments of physical capacity improvement [52].

### 4.2. Relationship of Peak Oxygen Uptake Improvement and Mitofusin1 Level

The increase in Mfn 1 levels in the plasma of patients who complete the IAP is interesting but difficult to interpret. It is not currently possible to link this increase to what is potentially going on in patients’ muscle or other tissues. Mitochondria are constantly being broken down and re-synthesised in energetically active cells including heart, muscle and brain and it has been recently demonstrated that plasma also contains high levels of mitochondria. Some appear to be functional [55] with others are released as a result of cell stress [56]. Mitophagy which drives the break -down of mitochondria and their subsequent recycling is an emerging area of mitochondrial biology with multiple types of mitophagy systems [57]. With most studies carried out in relatively inactive cell lines in vitro it is difficult to know how a highly active tissue like heart and muscle deals with the high demand for mitochondrial turnover. Extracellular vesicles are found at high levels in plasma (reviewed in [58] and contain various cargos including fragments of mitochondria [59] reviewed in [60]. You could speculate that in a very active tissue cells could get rid of larger mitochondrial fragments more rapidly in an extracellular vesicle system than by mitophagy. This could link to the high numbers of mitochondria found in plasma in previous studies and might explain increased levels mitofusin 1 in the plasma of the CFS patients following an IAP. High Mfn1 plasma levels could be linked to either the beneficial or detrimental effects of increased levels of activity in CFS patients. On the positive side, muscle being induced by exercise to form a more network efficient mitochondrial structure could result in an increase in mitochondrial associated Mfn1 debris, expelled to plasma would be linked to enhanced mitochondrial turn over and activity. Detrimentally, high levels of Mfn1 may reflect increased levels of mitochondrial fragmentation linked to induced mitochondrial stress associated with exercise in ME/CFS which may relate to further dysfunction in the future. Without a healthy control group completing the IAP programme it is difficult to know if this increase is just linked to increased exercise or is specific to the CFS group.

Both Mfn1 and Mfn2 play role in the mitochondrial fusion [61]. Mfn1 but not Mfn2 was able to decrease mitochondrial fusion in rodents’ skeletal muscle [62]. In a recent rodent-based study, endurance training has been shown to lead to an increase of Mfn1 expression in liver, while a decrease in the sedentary control group with high-fat diet was noted [63].

Exercise training appears to regulate both mitochondrial fusion and fission processes. Seven training sessions of high intensity interval training (HIIT) have been shown to progressively elevate protein content of Mfn1 in human skeletal muscle [64]. In addition, 24 h following a single training session of cycling exercise enhancement of both Mfn1 and Mfn2 mRNA content in human skeletal muscle was observed [21]. However, results of other study contrasts with those discussed above. In a recent study, the effects of a high-intensity interval training (HIIT) program with a progressive increase in intensity on mitochondrial function was assessed. Participants were healthy and recreationally undertaking a physical exercise program before taking part in the study. During the fourth week of the program subjects reached 5 HIIT sessions per week at 8 min intervals with intensity of 90% of VO_2_max and 3 min of rest between intervals [65]. After the fourth week disturbances in mitochondria function and impaired glucose homeostasis were observed [65]. It could be speculated that in the case of some of patients in the current study who were unable to complete this was related to the intensity of IAP. Interestingly, both IAP and GET in the PACE trial [51] programmes consisted of five training sessions per week. In contrasts, five HIIT sessions per week has been used to intentionally induce overreaching in healthy participants [65]. We suggest that further studies should consider a personal medicine approach to distinguish whether activity could be considered at all in individual CFS patients. If considered, appropriate the characteristics of a physical activity program need to be considered in terms of frequency, sessions intensity, duration and the type of exercise (interval vs. continuous, endurance vs. strength training, etc.) that are more likely to benefit each particular CFS patient. It seems unlikely that one regime will be appropriate for all those with CFS and for many patients perhaps only pacing would be appropriate at a particular time [66]. Moreover, more studies on predictors of the adverse effects of physical activity/exercise and its underlying pathological mechanism in CFS patients are needed.

### 4.3. The Relationship between of Submax VO_2_ Improvement and ANS Responsiveness

In the current study, improvement in submax VO_2_ was related to a decrease in sympathovagal balance in response to the HUTT. Only one patient from 34 was able to reach 80% HRmax intensity during the last training session in a programme. Therefore, the general tendency in our patients was the inability to reach 80% of calculated HRmax even after 15 weeks of training program. Chronotropic intolerance was noted in the current sample and is in line with previous studies on CFS patients [67,68]. A previous study noted that an aerobic exercise training program might lead to improvement in ANS functioning [11]. On the other hand, CFS seems to be an exceptional disorder, in which rapid and dramatic deterioration of symptoms might be induced by physical exercise in patients with post-exertional malaise (PEM) [69]. Poor recovery of diastolic blood pressure and reduced parasympathetic reactivation during recovery from exercise have been previously associated with the pain increases following exercise which are part of the PEM response seen in CFS, evidencing a possible link between ANS dysfunction and PEM [70].

In a study published in 2006, a positive correlation between the measurement of vagal nerve activity and VO_2_max was reported for the first time [71]. This positive relationship between HRV and exercise performance is consistent with a large number of previous studies that link variation in heart period [72], total HRV spectral power [73], and HRV triangular index (HRVI) [74] with VO_2_max. In addition, cross-sectional studies suggest that higher cardiopulmonary fitness is associated with increased vagal nerve activity [75,76]. The training response was correlated with age (r = 0.39) and with the values of the high-frequency spectral component (HF) of the RR intervals (HF power) analysed during the 24-h recording (r = 0.46), during the day (r = 0.35) and strongest at night (r = 0.52). These data show that the function of the autonomic cardiovascular system is an important determinant of the response to aerobic training in sedentary men. High vagal activity prior to undertaking a training program is associated with an improvement in aerobic capacity as a result of a training program of aerobic exercise in healthy, sedentary individuals [77]. Night-time HF power at baseline was the most effective predictor that explained 27% of the variance in VO_2_max improvement with the training program used.

The mechanisms underlying the relationship between ANS and physical training program response remains speculative. Consistent with the high inter-individual and intra-individual variability in training response to exercise, there was also a wide inter-individual variability in the autonomic regulation of the cardiovascular system in healthy subjects, as measured by indices of HR variability [78]. Genetic factors can account for a large proportion (about 20%) of the inter-individual variability in HR [79,80], while demographic and other factors, including blood pressure, blood cholesterol, heart size, body mass index and smoking account, explain only a small part (about 10%) of the variability of the autonomous regulation [80]. It is also possible that there is a mechanistic relationship between the function of the vagus nerve and the response to a training program. In people with optimal vagal function, the cardiovascular system may be better able to adapt to a variety of external stimuli, such as exercise. This adaptive ability can improve overall cardiovascular fitness after regular physical training and thus improve aerobic capacity [77].

### 4.4. Study Limitations

We have noted a considerable withdrawal rate (35 from 69 patients) from the intervention which was mainly due to the development of PEM. Sixteen patients were unable to complete CPET at baseline and therefore we were unable to incorporate this subgroup in all comparisons. Mitofusins level were analysed using ELISA, which has limits in its precision of measurement level [81]. Moreover, some samples were frozen longer than others, as patients did not start the physical activity program simultaneously. Recently, it has been shown that time of samples being frozen could confound the results [82]. A significant limitation for this study was that PEM was not measured. Moreover, further studies should use questionnaires to examine effects of therapy on potential CFS comorbidities such as anxiety and depression. Due to the relatively small sample size, results on effects of IAP should be replicated in further studies. Additionally, no control group was applied in the above study, limiting the conclusions that can be drown from this study. Future research study should incorporate daily or weekly questionnaires assessing PEM in ME/CFS patients undergoing aerobic exercise program.

Future studies on mechanism underlying PEM should consider a crossover-type trial of a supervised physical activity programme with low load for 12 weeks followed by 12 weeks of high load, to ensure that individuals who take part in the study could be their own controls.

## Figures and Tables

**Table 1:** Patients’ characteristics before Individualised Activity Program (IAP).

Variable (Unit)	Mean (SD) before IAP (*n* = 34)
Age (years)	37.06 (7.9)
BMI (kg/m^2^)	24.52 (3.2)
FFM (kg)	54.45 (9.7)
Fat (%)	25.04 (6.6)
HADS_A_(points)	10.30 (3.8)
HADS_D_(points)	8.76 (3.2)
BDI_(points)	17.97 (9.1)
HR_(bpm)	69.75 (7.9)
sBP_(mmHg)	116.98 (12)
dBP_(mmHg)	79.45 (10.8)
CI (l/min/m^2^)_	3.54 (0.9)

BMI—body mass index, FFM—free-fat mass, HADS_A—Hospital Anxiety and Depression Scale (anxiety score), HADS_D—Hospital Anxiety and Depression Scale (depression score), BDI—Beck Depression Inventory, HR—hear rate, sBP—systolic blood pressure during rest, dBP—diastolic blood pressure during rest, CI—cardiac index.

## Data Availability

Individual data is available from the corresponding author S.K. on request.

## Supplementary Materials

The following are available online at https://www.mdpi.com/article/10.3390/jcm10071542/s1, Table S1: Comparison of results before vs. after IAP in fatigue scales; Table S2: Comparison of results before vs. after IAP in body composition; Table S3: Comparison of results before vs. after IAP in autonomic nervous system functioning; Table S4: Comparison of results before vs. after IAP in mitofusins level; Table S5: Comparison of results before vs. after IAP in CPET results.
