# Supplementary material for: Relationship between Cardiopulmonary, Mitochondrial and Autonomic Nervous System Function Improvement after an Individualised Activity Programme upon Chronic Fatigue Syndrome Patients
Source: J Clin Med. 2021 Apr 6;10(7):1542. doi: 10.3390/jcm10071542 (PMC8038762; doi:10.3390/jcm10071542)

Table S1. Comparison of results before vs after IAP in body composition.

Variable [unit]	Mean (SD) before	Mean (SD) after	Original P value	FDR Adjusted P value	Significant using an FDR of 0.05?
BMI	24.52 (3.2)	24.28 (3.2)	0.90	0.90	No
Body fat [%]	25.04 (6.6)	24.78 (7.2)	0.63	0.81	No
FFM [kg]	54.45 (9.7)	54.58 (10.5)	0.87	0.90	No

BMI – Body Mass Index, FFM – Free-fat Mass

Table S2. Comparison of results before vs after IAP in fatigue scales.

Variable [unit]	Mean (SD) before	Mean (SD) after	Original P value	FDR Adjusted P value	Significant using an FDR of 0.05?
CFQ [points]	26.12 (3.49)	9.68 (6.22)	0.0000004	0.000004	Yes
FSS [points]	48.91 (8.95)	40.15 (10.87)	0.00005	0.0004	Yes
FIS [points]	93.59 (24.86)	61.68 (32.96)	0.0000001	0.000002	Yes

CFQ – Chronic Fatigue Questionnaire, FSS – Fatigue Severity Scale, FIS – Fatigue Impact Scale

Table S3. Comparison of results before vs after IAP in CPET results.

Variable [unit]	Mean (SD) before	Mean (SD) after	Original P value	FDR Adjusted P value	Significant using an FDR of 0.05?
RER peak	1.14 (0.1)	1.15 (0.1)	0.83	0.90	No
HR peak [1/min]	171.12 (19.9)	172.53 (18.2)	0.76	0.88	No
VO_2 peak [ml/kg/min]	30.3 (5.6)	31.79 (5.45)	0.047	0.09	No
Load/kg peak [W/kg]	1.85 (0.3)	342.09 (0.5)	0.005	0.01	Yes
RER AT	0.98 (0.04)	1.00 (0.03)	0.009	0.02	Yes
HR AT [1/min]	144.21 (16.4)	153.03 (21.6)	0.01	0.02	Yes
VO_2 AT [ml/kg/min]	22.35 (4.8)	24.88 (4.8)	0.003	0.01	Yes
Load/kg AT [W/kg]	1.31 (0.3)	1.61 (0.4)	0.00007	0.0004	Yes

VO_2/kg (ml/min/kg) – oxygen consumption, RER – ratio of VCO_2 to VO_2, HR [bpm] – heart rate, Load [W/kg] – load divided by body mass of participant, peak – values obtained during maximal intensity exercise, AT – values obtained during anaerobic threshold

Table S4. Comparison of results before vs after IAP in mitofusins level.

Variable [unit]	Mean (SD) before	Mean (SD) after	Original P value	FDR Adjusted P value	Significant using an FDR of 0.05?
mitofusin1 [ng/mL]	0.22 (0.26)	0.33 (0.27)	0.002	0.007	Yes
mitofusin2 [ng/mL]	5.51 (1.51)	8.05 (2.73)	0.00005	0.0004	Yes

Table S5. Comparison of results before vs after IAP in autonomic nervous system functioning.

Variable [unit]	Mean (SD) before	Mean (SD) after	Original P value	FDR Adjusted P value	Significant using an FDR of 0.05?
LFnu-RRI in supine [%]	56.81 (14.9)	60.67 (16.1)	0.66	0.81	No
HFnu-RRI in supine [%]	43.19 (14.9)	39.33 (16.1)	0.66	0.81	No
LF/HF-RRI in supine	1.91 (2.0)	2.09 (1.3)	0.13	0.24	No
HUTT LFnu-RRI [%]	9.87 (11.9)	7.63 (11.1)	0.22	0.32	No
HUTT HFnu-RRI [%]	-9.87 (11.9)	-7.63 (11.1)	0.22	0.32	No
HUTT LF/HF-RRI	1.63 (2)	1.56 (2.8)	0.18	0.30	No

LF, low frequency. HF, high frequency. LFnu, HFnu, frequencies calculated in normalized units. RRI, R-R interval

Figure S1. Heatmap of Spearman's correlation between outcomes of the study. A) correlation of values before IAP, b) correlation of values after IAP, c) correlation of difference before-after IAP.

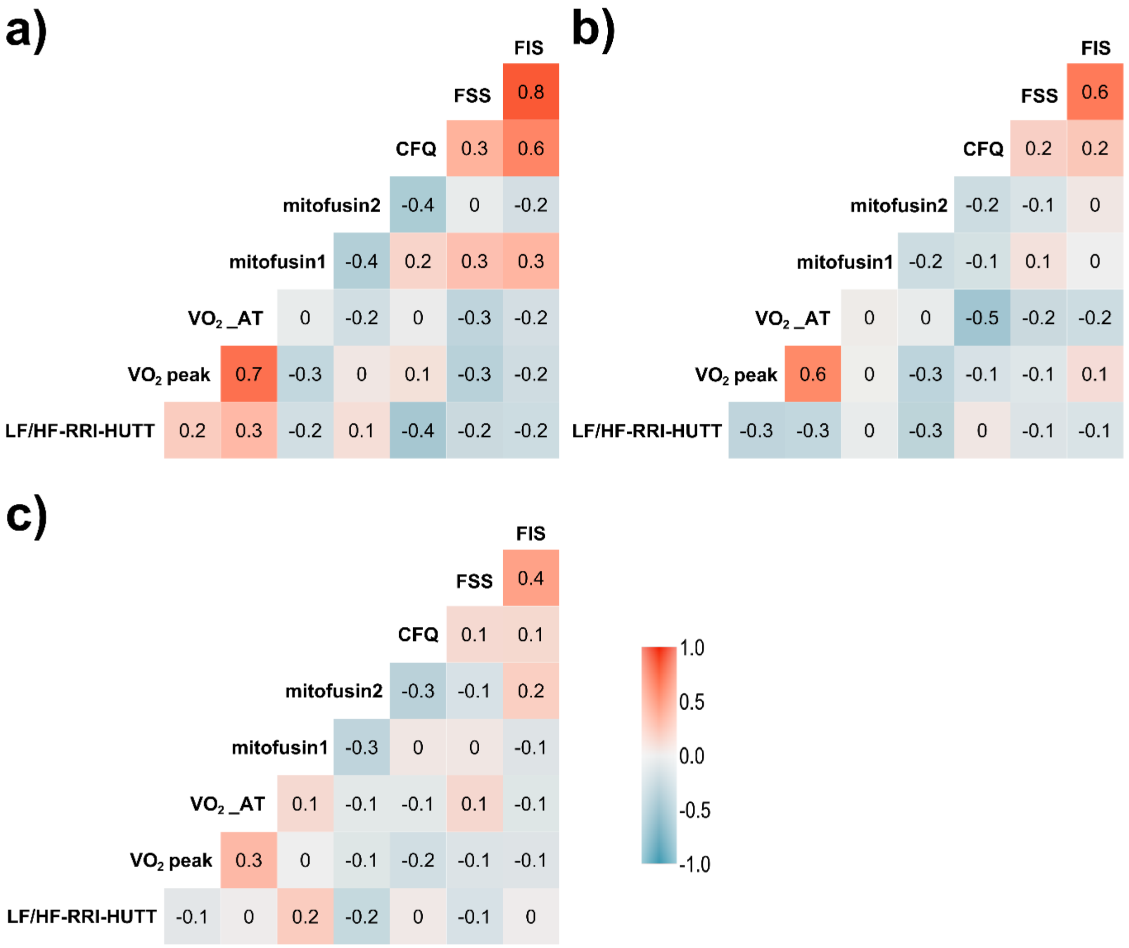

Supplement: Supplementary file 1 [file jcm-10-01542-s001.pdf]
